# Combination Therapy Approaches to Enhance the Efficacy of ERV-Targeting Vaccines in Cancer

**DOI:** 10.1158/2326-6066.CIR-24-1192

**Published:** 2025-05-19

**Authors:** Maria Gracia-Hernandez, Maria del Mar Maldonado, Jeffrey Schlom, Duane H. Hamilton

**Affiliations:** Center for Immuno-Oncology, Center for Cancer Research, National Cancer Institute, National Institutes of Health, Bethesda, Maryland.

## Abstract

Endogenous retroviruses (ERV) are the genetic remnants of retroviruses in which proviral sequences integrated into germline cells of our ancestors. Although the vast majority of ERV sequences have accumulated mutations over the course of human evolution, some still contain open reading frames encoding full-length retroviral proteins. These sequences are typically epigenetically silenced in healthy adult human tissues. However, epigenetic dysregulation in cancer results in aberrant expression of ERVs in multiple cancer types. Therefore, ERVs represent a class of attractive therapeutic targets in cancer due to their immunogenicity and high expression in cancer cells compared with healthy tissues. In this review, we summarize the roles of ERVs in cancer and their immunogenicity, highlight the most recent advances in ERV-targeting strategies, discuss their challenges, and examine potential combination approaches that could further enhance the antitumor efficacy of ERV-targeting vaccines.

## Introduction

**Figure 1.**
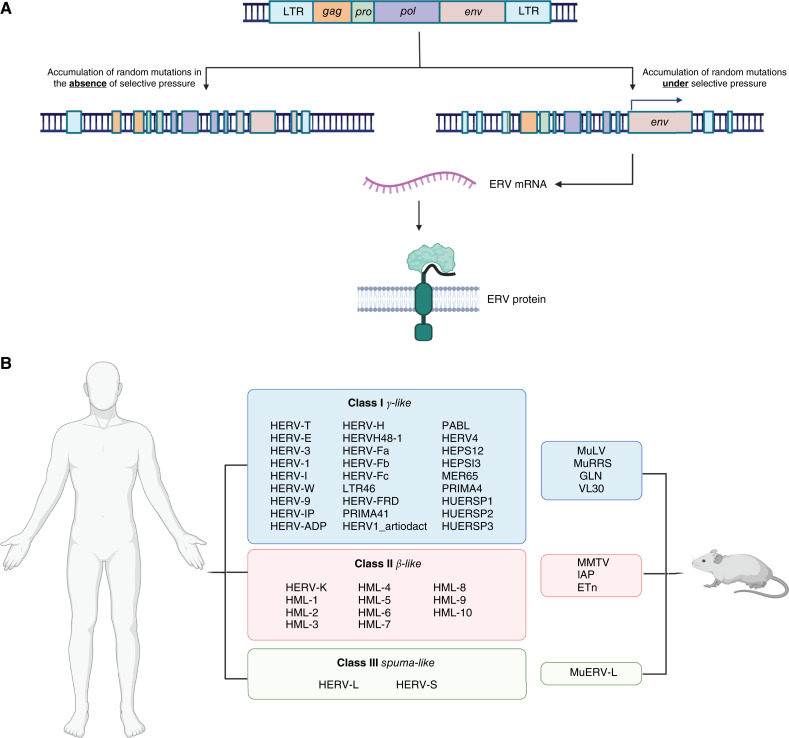
Maintenance of endogenized viral genomes. **A,** An intact ERV sequence is composed of *gag*, *pro*, *pol*, and *env* genes flanked by two LTRs. The vast majority of proviral genomes are highly fragmented and do not encode full-length proteins. However, a small number of proviral genomes are still able to encode full-length proteins following selective pressure. **B,** ERVs are classified into three classes, class I (γ-like), class II (β-like), and class III (spuma-like; refs. 2–4). Panel A image modified from previous literature (Song et al., ref. 2, reprinted with permission from Springer Nature). Created with BioRender.com. Maldonado Montalban, M. (2025) https://BioRender.com/c47u762.

Retroviruses are unique among RNA viruses as their replication requires integration into the genome of their host. Although infection and integration usually occur in somatic cells, on rare occasions they may occur in germline cells. In these instances, the provirus can be transmitted to subsequent generations through a process called vertical transmission. Endogenous retrovirus (ERV) sequences are thought to comprise 8% of the modern human genome (1). ERV proviruses retain the retrovirus structure and are composed of *gag*, *pro*, *pol*, and *env* genes flanked at either side by long terminal repeats (LTR) and can be classified into three classes (γ, β, and spuma-like) based on their relationship to other retroviruses (Fig. 1; ref. 5). The vast majority of ERV sequences integrated into the genome thousands of years ago and have accumulated mutations over the course of human evolution. These mutations result in the fragmentation and/or inactivation of the provirus, and despite their abundance, it is estimated that approximately 100 ERVs conserve an intact or nearly intact provirus that could theoretically produce intact viral particles. However, multiple studies have not been able to detect ERV-derived viruses in human samples (6, 7). Although mutated proviruses may not be capable of producing viral particles, several encode full-length proteins.

Expression of ERV-encoded proteins is tightly regulated in healthy tissues epigenetically (8). One of the hallmarks of cancer is epigenetic dysregulation (9), which may result in the aberrant expression of ERV-encoded proteins in multiple tumor types, with human ERV-K (HERV-K) being the most common (10). The immune system is not ignorant to this aberrant expression of ERVs in carcinomas. Several studies have demonstrated ERV-reactive T cells and ERV-specific antibodies in patients with breast, ovarian, and kidney carcinomas (10–15). This demonstration of immunogenicity as well as the high expression of many ERVs in carcinoma cells, as compared with adjacent healthy tissues (16), makes ERVs an attractive therapeutic target for cancer immunotherapy.

In this review, we summarize the current literature reporting promising preclinical studies targeting ERV sequences using various therapeutic modalities, such as antibodies, cellular therapies, and therapeutic cancer vaccines. Furthermore, we provide potential combination strategies of ERV-targeting agents with other immuno-oncology therapies such as epigenetic modifiers, immune checkpoint inhibitors (ICI), and cytokines. In addition to describing characteristics that make them targetable, we also highlight potential challenges to their clinical development.

## ERV Expression in Healthy and Cancer Tissues

**Figure 2.**
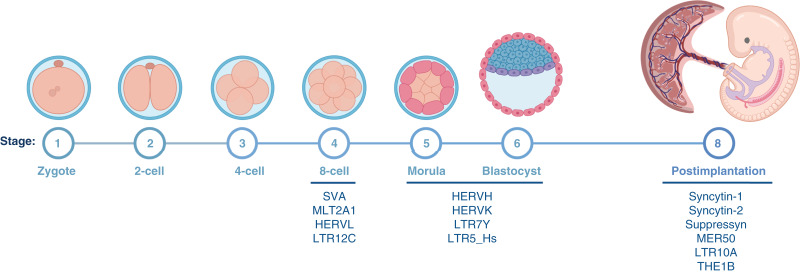
ERV proteins with essential roles during human embryogenesis. Image modified from previous literature (Reprinted Wang et al., ref. 26, © 2024, with permission from Elsevier; used with permission of Springer Nature BV from Dopkins et al., ref. 27, © 2024, permission conveyed through Copyright Clearance Center, Inc.). Created with BioRender.com. Maldonado Montalban, M. (2025) https://BioRender.com/r46z436.

The majority of ERV-encoded open reading frames (ORF) are not known to play any role in healthy tissues, and their expression is actively silenced (17–19). However, a small subset of ERV ORFs has been reported to play critical roles in normal cellular functions and processes, such as embryogenesis, immune regulation, and aging (19–23). The required function of ERVs in healthy tissues suggests the presence of a dynamic, selective regulation of expression. For example, human ERV sequences such as HERV-L, HERV-H, and HERV-K, among others, are dynamically expressed and regulated (activated or silenced) during embryogenesis, placentation, placental evolution (24, 25), and somatic development (Fig. 2). Aberrations in their regulation can lead to pathologic changes (28, 29). In addition, it was recently shown that ERV-derived ORF expression is thought to protect trophoblasts from infection by exogenous retroviruses (26, 30, 31). Overall, the majority of ERV sequences are not transcriptionally active in healthy tissues; however, some have been reported to play essential cellular functions.

In most cases, the silencing of ERV sequences in healthy adult tissues is thought to be achieved via promoter methylation. As global DNA hypomethylation is a common occurrence in most cancers, it is not surprising that many ERV-encoded proteins are aberrantly expressed in tumor tissues (32, 33). In support of this theory, Gimenez and colleagues (16) performed an HERV-targeted microarray assay using testicular cancer samples and found that the LTRs of six HERV-W loci that were upregulated in tumors had methylation levels ranging from 0% to 30% compared with 82% to 100% in healthy tumor-adjacent tissues. This reduction in methylation associates with enhanced expression of ERV sequences. In addition to promoter methylation, recent publications have highlighted the role of regulators of chromatin structure in the silencing of ERV sequences, such as SETDB1 and TRIM28. SETDB1 is a histone 3 lysine 9 (H3K9) methyltransferase that has been shown to silence ERV sequences by maintaining trimethylation of H3K9 and heterochromatin status (34). TRIM28 is a transcriptional corepressor that is recruited to ERV sequences in the genome and forms a complex with SETDB1 and other proteins to actively silence ERV transcript expression (35, 36). In mouse tumor models, loss of SETDB1 results in expression of ERV sequences, which induce ERV-derived type I IFN responses, which associates with enhanced lymphocyte infiltration, reduced tumor growth, and enhanced responsiveness to radiotherapy (37, 38). Conversely, the SETDB1–TRIM28 complex has been reported to suppress antitumor immunity (39). In a recent publication, Alcazer and colleagues (40) were not only able to distinguish between healthy hematopoietic and acute myeloid leukemia (AML) cells but also to classify AML into distinct subtypes with differing prognosis based upon their chromatin structure and ERV expression. These observations demonstrate the interdependence of chromatin architecture and ERV expression in cancers.

It has been reported that an average of 2,736 ERV sequences are expressed in each tumor cohort evaluated (pancreatic, liver, clear-cell kidney, ovarian, bladder, lung, and breast carcinomas), with kidney cancers having the highest number of expressed ERVs and liver cancers having the lowest number (bioRxiv.2024.02.07.579350). The authors found that the most expressed ERV families across these cohorts were HERVH, HERVL, ERVLE, ERV316A3, MER4, MER41, and ERVLB4.

In general, ERV sequences have been found to be highly expressed in hematologic malignancies (i.e., leukemias and lymphomas) as well as in prostate, ovarian, bladder, lung, colon, and liver cancers (41). In a recent review, Müller and colleagues (10) surveyed the literature and summarized ERV-derived proteins detected in samples of patients with cancer and/or cancer cell lines (Table 1). Among all ERV sequences, HERV-K is the most frequently expressed ERV across different cancer types, and it is expressed in germ cell tumors, neurologic cancers, melanoma, and prostate cancer, among others. After HERV-K, the other most common cancer-associated ERV types are HERV-H, HER-W, and HERV-R (42).

**Table 1.** ERV protein expression in multiple human cancer types.

Cancer type	ERV type
Germ cell tumors	HERV-K
Neurologic cancer	HERV-K
Skin cancers	HERV-K, HERV-W, and HERV-H
Prostate cancer	HERV-K
Hematologic malignancies	HERV-K, HERV-W, HERV-H, HERV-E, and HERV-FRD
Mammary cancers	HERV-K
Gastrointestinal cancers	HERV-K, HERV-W, HERV-H, HERV-3, and HERV-FRD
Gynecologic cancers	HERV-W, ERV-3, HERV-K, HERV-FRD, HERV-E, ERVMER34-1, and HERV-V
Urological cancers	HERV-E, HERV-K, and HERV-W
Lung cancers	HERV-K
Pancreatic cancers	HERV-K and HERV-H
Endocrine cancers	HERV-W
Sarcomas	HERV-K

Table adapted from Müller and colleagues (10).

The CancerHERVdb database provides an overview of the literature reporting the expression of specific ERV sequences across different types of cancer, and it serves as a tool to identify ERV sequences that could potentially be targeted in cancer. To illustrate, this database shows that a high percentage of samples from patients with cancer express different ERVs, such as HER-V, HERV-K, and HERV-H LTR–associating protein 2 (*HHLA-2*), among others (summarized in Table 2; ref. 43).

**Table 2.** Percentages of ERV sequences expressed in different cancer types according to CancerHERVdb database (43).

ERV type	Cancer type	ERV-positive samples	Refs.
HERV-W env	Colorectal cancer	84.5%	(44, 45)
	Liver cancer	81.55%	(46)
	Urothelial cell carcinoma	75.6%	(47)
HHLA-2	Pancreatic ductal adenocarcinoma	71.9%	(48)
	Oral squamous cell carcinoma	68.66%	(49)
	Gastrointestinal cancer	61.1%	(50)
	Intrahepatic cholangiocarcinoma	54.59%	(51)
	Hepatocellular carcinoma	50.99%	(52, 53)
	Lung cancer	46.83%	(54)
	Clear cell renal cell carcinoma	40.4%	(55)
HERV-K Gag	Prostate cancer	18%	(43)
HERV-W env and pol, HERV-T pol, HERV-Rb pol, HERV-K pol, and HERV-E gag and pol	Bladder cancer	72.7%–100%	(43)

## Role of ERVs in Cancer Progression

It has become increasingly apparent that ERV-encoded proteins may play active roles in tumor progression. ERV-derived Env proteins have immunosuppressive properties and play important roles in cancer development, progression, and resistance to therapies (56). Expression of the ERV-encoded Env protein *ERVH48-1* (suppressyn) has been shown to promote the proliferation of prostate cancer cells and resistance to doxorubicin (57). Furthermore, additional Env proteins such as those of *ERV3-1*, *ERVW-1*, and *ERVFRD-1* have tumor suppressor roles, whereas *ERVV-1*, *ERVK13-1*, and *ERVMER34-1* may promote oncogenesis in breast cancer (58).

Additionally, ERVs actively dampen the generation of effective antitumor immunity. For example, the ERV-derived immune checkpoint *HHLA-2* is expressed in many types of human cancers such as melanoma, breast, colon, pancreatic, among others, and high *HHLA-2* expression is associated with poor prognosis (59). *HHLA-2* has immunosuppressive properties as it interacts with *KIR3DL3* to inhibit CD8^+^ T-cell and NK-cell function and induce resistance to tumor-cell killing (60). Additionally, overexpression of ERVE-4 and HERV 4700 was observed in patients with clear cell renal cell carcinoma (ccRCC) who did not respond to anti–PD-1 therapy (61). Ng and colleagues (62) found that HERV-targeting B-cell responses can be amplified by ICIs in mouse models and patients with lung adenocarcinoma, and ERV expression can predict the outcome of ICIs in these patients. In contrast, it has also been observed that expression of other ERV sequences may associate with responsiveness to immunotherapy. In non–small cell lung cancer, patients who have high MER4 expression associate with better progression-free survival and overall survival as these patients also display inflammatory gene signatures and demonstrate improved responsiveness to ICIs (63).

## ERV Expression and Response to Immunotherapy

Currently, researchers are evaluating the relationship between ERV sequence expression signatures (comprising multiple ERVs) and resistance or response to immunotherapy. Panda and colleagues (64) investigated the association between ERV expression in tumors and local immune checkpoint activation and responses to ICIs. They reported that tumors expressing high levels of ERV sequences had increased immune cell infiltration, upregulated checkpoint pathways, and higher CD8^+^ T-cell fractions compared with ccRCC tumors with low ERV expression. They observed similar trends in colon, neck squamous cell, and breast tumors. Furthermore, the authors discovered that ERV3-2 expression was significantly higher in anti–PD-1/PD-L1 responders compared with nonresponders in patients with metastatic ccRCC (mccRCC). Likewise, Zhou and colleagues (65) evaluated three clinical trials in which patients with ccRCC were treated with anti–PD-1 and found an ERV signature comprising nine ERVs that could stratify patients into high- or low-risk categories. Patients in the low ERV risk category had higher CD8^+^ T-cell infiltration, higher probability of survival, and better prognosis than patients in the high ERV risk category. Therefore, this ERV signature may serve as a predictive and prognostic biomarker for patients with advanced ccRCC who receive anti–PD-1 therapy. Lastly, another group identified a relationship between ERV abundance, immunogenicity, and epigenetic dysregulation in metastatic breast, colorectal, and pancreatic ductal adenocarcinoma tumors (66). The authors reported a positive correlation between tumors that express high levels of ERV sequences with gene signatures of immunogenicity and autonomous antiviral responses in the three tumor types evaluated. Specifically, they found significantly strong positive correlations between ERV sequence expression and genes associated with neutrophils, T cells, and IFN signaling. Interestingly, the authors also found that colorectal and pancreatic tumors that had a viral mimicry phenotype had increased expression of *TET2*, a DNA demethylation gene (67), thus suggesting that aberrations in DNA methylation may play an important role in the expression of ERV sequences and in gene signatures of immunogenicity and viral mimicry.

In summary, the high expression patterns of ERV sequences in numerous cancer types compared with healthy adult tissues, in addition to the critical roles that ERVs play in cancer progression and resistance to conventional therapies and immunotherapies, make ERVs potential targets for cancer immunotherapy.

## Immunogenicity of ERVs

Although most ERV proviral sequences have accumulated mutations preventing the production of viral particles, some encode full-length ORFs that encode retroviral proteins (68). In cancers, ERV-derived antigens can be generated via multiple mechanisms. ERV-derived immunogenic epitopes can be derived via canonical ERV ORFs, chimeric endogenous retroelement–exon tumor antigens (69), or noncanonical splicing junctions between exons and transposable elements (70). The latter two mechanisms generate aberrant ERV-derived proteins that are uniquely expressed in tumor cells and absent from healthy tissues. ERV-specific immunity, detected against ERV proteins expressed by each of the three above processes, has been identified in patients with cancer (summarized in Table 3; refs. 78, 79).

**Table 3.** Immune responses against ERVs in patients with cancer.

Response	ERV type	Cancer type	Refs.
ERV-specific T cells	HERV-K ENV	Ovarian cancer	(12)
	HERV-K ENV	Breast cancer	(11)
	HERV-K GAG and POL	Breast cancer	(71)
	HERV-derived peptides with sequences not registered in public protein databases	Kidney cancer	(72)
	HERV-K GAG	Seminoma	(73)
	HERV-K MEL	Melanoma	(74)
	HERV-E	Kidney cancer	(75)
ERV-specific antibodies	HERV-K ENV	Breast cancer	(15)
	HERV-K	Ovarian cancer	(12)
	HERV-K10 GAG	Seminoma	(76)
	HERV-K GAG/ENV	Germ cell tumors	(77)

Multiple research groups have demonstrated that ERV-derived peptides are processed and presented by the immunopeptidome, and these presented ERV epitopes can expand antigen-specific T cells with cytotoxic activity and high avidity against tumor cells or organoids (12, 71, 72). In addition, several reports have shown that ERV-specific CD8^+^ T-cell responses directed against multiple ERV-derived proteins (i.e., gag, rec, env, and reverse transcriptase) have been detected in the blood of a large proportion of patients diagnosed with different types of cancer (78). In one such study, Saini and colleagues (80) found that half of the patients with hematologic malignancies who were included in the study had specific T cells against ERV peptides derived from *HERVE-3*, *HERVH-5*, and *HERVW-1*. In addition, patients with melanoma have HERV-K–specific T cells in circulation, and these are absent in healthy donors as HERV-K is a tumor-associated antigen that is expressed by melanoma cells but not normal cells (74, 81). Besides inducing a T-cell response, ERV-derived proteins may also induce humoral responses. As such, HERV-K immunoreactive antibodies have been found in the serum of patients with ovarian cancer (12), seminoma (76), germinal cell cancer (77), and breast cancer (15).

Although there are several studies evaluating the presence of ERV-specific T cells and demonstrating their ability to kill cancer cells, relatively few studies have identified specific ERV-derived T-cell epitopes and characterized the T-cell clones generated (11, 13, 73–75, 82). One such study, performed by Bonaventura and colleagues (71), identified nominal CD8^+^ T-cell epitopes from conserved Gag and Pol HERV-K motifs in patients with triple-negative breast cancer and found that 7 of 11 patients had T-cell receptors (TCR) with specificities for these HERV-K epitopes.

## Potential Challenges for Targeting ERVs

### Immunosuppression mediated by ERVs

**Figure 3.**
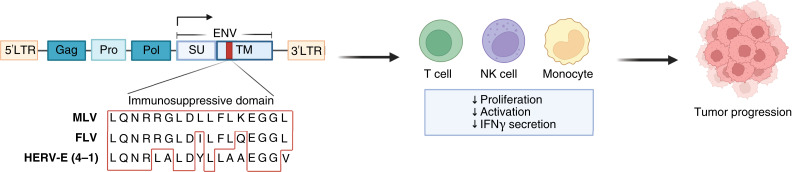
The ISD within the retroviral ENV protein is conserved among ERVs from different species, as seen in murine leukemia virus (MLV), feline leukemia virus (FLV), and human ERVs, such as HERV-E clone 4-1. Expression of the retroviral ENV protein increases immunosuppression by inhibiting lymphocyte proliferation and decreasing activation of monocytes, T cells, and NK cells that could further contribute to tumor progression. Image modified from previous literature (Cianciolo et al., ref. 86. Reprinted with permission from American Association for the Advancement of Science). Created with BioRender.com. Maldonado Montalban, M. (2025) https://BioRender.com/w77o051.

It has been well established that one mechanism by which retroviruses promote their propagation is by actively dampening the immune system within infected hosts. Multiple studies have described the presence of a highly conserved immunosuppressive domain (ISD) within the retroviral Env protein. This ISD can suppress proliferation and activation of macrophages, lymphocytes, and monocytes (83–86). As such, tumor cells expressing envelope proteins containing the ISD may also utilize a similar mechanism to evade immune surveillance (Fig. 3). A study by Mangeney and colleagues (87) demonstrated that expression of the full-length HERV-H Env protein in a murine carcinoma cell line enabled tumor growth when cells were implanted in either allogeneic or syngeneic immunocompetent mice. Other examples of ERV Env proteins containing immunosuppressive regions include HERV-P(b) and HERV-V Env proteins (41). Thus, the initial challenge of ERV-targeting therapies resides in that they must generate an immune response strong enough to surmount the immunosuppression of the ISD of ERV Env proteins.

### Expression of ERVs in healthy human adult tissues

It is well known that several ERVs, such as HERV-K, are expressed in the pluripotent human stem cells in the placenta and may also be present in other healthy adult tissues (88–92). Burn and colleagues (93) characterized the expression of HERV-K (HML-2) provirus in healthy tissues sampled at autopsy using RNA sequencing datasets from the Genotype Tissue and Expression project. Following assessment of 54 different tissues corresponding to 948 donors, HERV-K provirus transcripts were found in every tissue evaluated, with higher levels being detected in the cerebellum, pituitary, testis, and thyroid (93). HERV-K–encoded *rec* and *np9* transcripts have also been detected in normal human tissues, such as the heart, brain, pancreas, and spleen, among others (94). However, these reports are balanced by negative protein staining in healthy adult tissues (95). Sacha and colleagues (95) also assessed this concern directly, by vaccinating rhesus macaques with consensus sequences of simian ERV-K Gag and Env and demonstrated that vaccination induced T-cell responses without vaccine-related pathologies. In humans, HERV-K‒specific T cells can be detected in the peripheral blood of patients with cancer without adverse effects (11, 12). Other studies have reported the presence of autoantibodies to HERV-K in patients with autoimmune and degenerative diseases such as systemic lupus erythematosus, rheumatoid arthritis, and Sjörgen syndrome, among other diseases (96, 97). However, there is a lack of consistency in autoimmune manifestations of autoantibodies targeting HERV-K in different diseases. Furthermore, autoantibodies targeting HERV-K have also been observed occasionally in healthy individuals (96).

Recently, a phase I clinical trial in patients with mccRCC assessed off-target toxicities following treatment with an HERV-E–directed therapy. In the study, patients were treated with adoptively transferred HERV-E TCR-transduced T cells without dose-limiting toxicities, off-target toxicities, or treatment-related deaths being reported at the end of the study (98). This lack of toxicity may depend on the ERV being targeted; thus, careful selection of the ERV target is key to ensure low or no expression in normal tissues and elevated expression in tumors to reduce off-target effects during treatment.

## Preclinical Studies Targeting Human ERVs

### Antibodies

ERV Env proteins are expressed on the cell surface, so one could potentially target them using specific antibodies. ERV-specific antibodies could neutralize ERV proteins and block their interaction with other proteins and mediate antibody-dependent cell cytotoxicity by NK cells, complement activation, or antibody-dependent cell phagocytosis by macrophages. Wang-Johanning and colleagues (99) developed mAbs against the HERV-K Env protein, which they found to be expressed in breast cancer cell lines and patient samples. They showed that treatment of breast cancer cells with these mAbs led to growth inhibition and apoptosis *in vitro*. Importantly, treatment with these mAbs significantly reduced tumor growth in xenograft models *in vivo*. Other groups have also shown that targeting the murine ERV envelope protein with mAbs reduces tumor growth, with some antibody clones curing mice and significantly enhancing survival of myeloid leukemia–bearing mice (100).

### ERV-specific T-cell transfer

Adoptive transfer of T cells engineered to express either chimeric antigen receptor (CAR) or TCR is an active area of investigation within the field of immuno-oncology. In preclinical models, Krishnamurthy and colleagues (81) engineered T cells to express a CAR that is derived from a mAb that targets HERV-K Env. These HERV-K Env–specific CAR-T cells were shown to have cytotoxic activity, recognize shed HERV-K Env, and reduce tumor growth of an HERV-K Env^+^ metastatic melanoma model. In addition, the same group tested the ability of HERV-K Env–specific CAR-T cells to inhibit breast cancer growth and metastasis in xenograft models (101). Only a handful of clinical studies targeting ERV^+^ cancers through cell-based therapies exist. Results from the first clinical trial evaluating TCR-engineered T cells targeting an ERV in patients with mccRCC (NCT03354390) showed that it is feasible to manufacture CD8^+^ HERV-E TCR-transduced T cells. These cells did not show any dose-limiting toxicities or off-target toxicities, proliferated *in vivo*, trafficked to metastatic sites, and resulted in partial responses in 7% of patients and stable disease in 29% of patients (98, 102).

### Vaccines in preclinical models

Several preclinical studies have tested ERV-targeted vaccines in various cancer models (Table 4). Neukirch and colleagues (104) targeted the envelope protein of melanoma-associated retrovirus, composed of p15E and gp70 subunits, by using vector-encoding virus-like particles. Vaccination increased secretion of IFNγ and TNFα by CD8^+^ T cells and reduced tumor progression in mice bearing CT26 murine colorectal tumors. Mice were also protected against rechallenge with 4T1 mammary tumors, which also express the target antigen gp70 (108). In a separate study, melanoma-associated retrovirus was targeted using virus-like particles encoding the envelope with a mutated ISD (Env ISDmut) as a surface target to improve the immunogenicity and efficacy of the vaccine (105). Vaccination resulted in increased multifunctional CD8^+^ T-cell responses and greater tumor control when compared with the vaccine targeting the nonmutated Env in the syngeneic CT26 murine colorectal tumor model. Additionally, combination with anti–PD-1 also showed higher curative efficacy in Env ISDmut‒vaccinated mice.

**Table 4.** Preclinical studies using ERV-targeting vaccines in cancer models.

Vaccine	Target	Vaccine platform	Tumor model	Combination strategy	Outcome	Refs.
MVA-Hkenv	HERV-K Env	Recombinant modified vaccinia virus Ankara	Murine Renca–expressing HERV-K ENV (RLZ-Hkenv)	N/A	Reduced pulmonary metastasis	(84)
					Prophylactic vaccination strategy protected against tumor development	
					Increased cytotoxicity against RLZ-Hkenv tumor cells	
MVA-Hkcon	HERV-K Gag	Recombinant modified vaccinia virus Ankara	Murine Renca–expressing HERV-K Gag (RLZ-HKGag)	N/A	Reduced tumor growth	(103)
					Reduced pulmonary metastasis	
Ad5-MelARV	MelARV Gag and Env (p15E and gp70)	Adenoviral vector-encoding VLPs	CT26 murine colorectal 4T1 murine mammary	Anti–PD-1	Reduced tumor growth and progression in mice	(104)
					Enhanced CD8 T-cell immunogenicity	
					Protected against rechallenge with different tumor type	
					Prophylactic vaccination strategy protected against tumor development	
Ad19a/64-ERV ISDmut	MelARV Gag and Env ISDmut	Adenoviral vector-encoding VLPs	CT26 murine colorectal 4T1 murine mammary	Anti–PD-1	Reduced tumor growth and progression in mice	(105)
					Enhanced T-cell immunogenicity	
					Cross-protection effects against a different tumor type	
					Increased effector CD8 T cells	
					Modest antibody responses	
ERV-PeptiCRAd	ERV LOC72520	PeptiCRAd platform complexed with FYLPTIRAV and TYVAGDTQV peptides	4T1 murine mammary	Anti–PD-1	Reduced tumor growth	(106)
					Increased IFNγ in restimulated splenocytes from vaccinated mice	
hAD19a/64 HERV-W	HERV-W Gag and Env (syncytin-1)	Adenoviral vector-encoding VLPs	Murine Renca–expressing HERV-W Env (Renca HERV-W Env)	N/A	Activation of antigen-presenting cells	(107)
					Increased specific T-cell responses	
					Prolonged survival	
					Extended survival	

Abbreviation: ISDmut, mutated immunosuppressive domain; MelARV, melanoma-associated retrovirus; Renca, renal carcinoma; VLP, virus-like particles.

Another study developed a customized oncolytic vaccine, ERV-PeptiCRAd, targeting ERV peptides previously identified through an MHC-I ligandome analysis in the 4T1 murine mammary tumor model (106). The PeptiCRAd vaccine platform consists of tumor peptides that are adsorbed onto an oncolytic viral capsid. Following intratumoral administration, the ERV-PeptiCRAd showed antitumor efficacy by reducing growth of 4T1 mammary tumors in mice. Interestingly, combination with anti–PD-1 did not increase the level of protection in this tumor model (106).

### Vaccines targeting human ERVs

Other studies have focused on developing vaccines targeting human ERVs such as HERV-K and HERV-W instead of murine retroviruses (84, 103, 107, 109). Kraus and colleagues (84, 103) tested vaccines targeting HERV-K Env and HERV-K Gag proteins in murine renal carcinoma cells genetically modified to express these targets. Both vaccines used the recombinant modified vaccinia virus Ankara as vaccine platforms. The HERV-K Env vaccine was able to reduce pulmonary metastasis *in vivo* and increase cytotoxicity against tumor cells. Moreover, prophylactic vaccination was able to protect mice against tumor development. Similarly, vaccination against HERV-K Gag also decreased tumor growth and pulmonary metastasis *in vivo* (103).

Recently, Skandorff and colleagues (107) targeted HERV-W (syncytin-1) by evaluating vaccines encoding either the nonmutated or mutated envelope ISD in a murine renal carcinoma model genetically modified to express HERV-W Env. The ISD of HERV-W is atypical due to its lack of immunosuppressive function that can be reversed to the immunosuppressive role following mutation (110). In this study, the antitumor effects of the wild-type HERV-W vaccine outperformed those observed with the ISD-mutated counterpart in this scenario. Vaccination with the wild-type HERV-W vaccine resulted in increased specific T-cell responses, enhanced activation of antigen-presenting cells, and augmented survival.

Together, these preclinical studies using ERV-targeting vaccines in tumor models have demonstrated immunogenicity of the targets and increased tumor control. Nonetheless, additional studies are needed to translate these preclinical findings into the clinical setting as these models are genetically engineered to express the target HERV and further improve antitumor efficacy.

## Combining ERV-Targeting Vaccines with Other Immuno-oncology Agents

**Figure 4.**
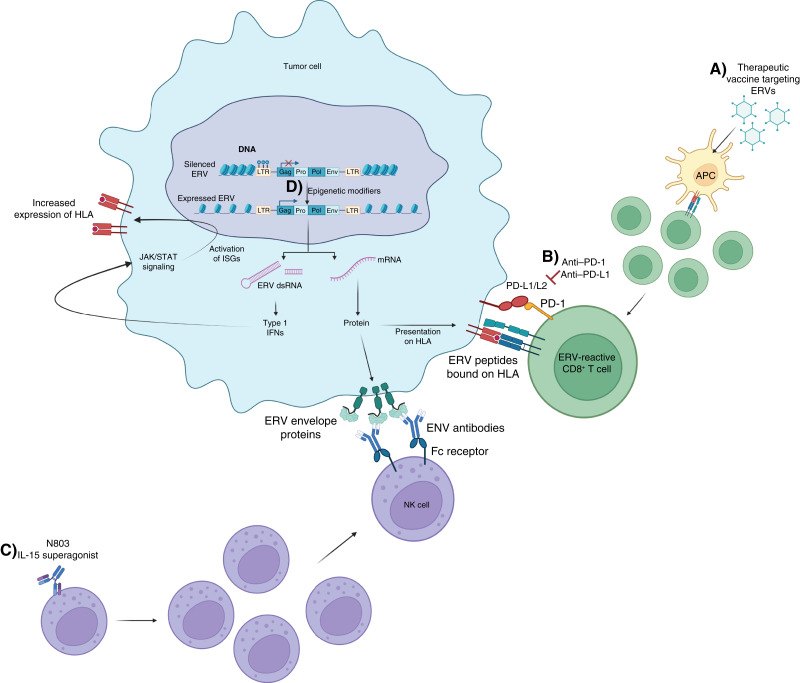
Potential combination approaches with therapeutic ERV cancer vaccines for targeting highly expressing ERV tumors. **A,** Vaccination with ERV-targeted vaccines using adenoviral vectors induces the expression of ERVs in antigen-presenting cells (APC), which in turn present the tumor antigen to CD8^+^ T cells and CD4^+^ T cells through MHC class I and MHC class II, respectively. **B,** ICIs such as anti–PD-1 further expand ERV-specific CD8 T cells by reducing immunosuppression in the TME. **C,** The use of additional immuno-oncology agents such as N803 could further enhance antitumor immunity by increasing T-cell and NK-cell activation. The presence of ERV antibodies in the TME could further promote antibody-dependent cytotoxicity by NK cells. **D,** Treatment with epigenetic modifiers, such as Aza, could further increase tumor cell visibility to immune surveillance by upregulating the expression of ERVs in tumor cells and promoting viral mimicry. Increased transcription of ERV double-stranded RNA (dsRNA) transcripts could trigger type 1 interferon responses and promote JAK/STAT signaling and activation of interferon-stimulated genes (ISGs), further increasing HLA expression in tumor cells. Created with BioRender.com. Maldonado Montalban, M. (2025) https://BioRender.com/i66w154.

One approach that could be used to further increase the efficacy of ERV-targeting vaccines in cancer models is to develop a combination strategy that adds other immuno-oncology agents as part of the treatment regimen. These combinations could potentially contribute to the antitumor immune response by increasing, expanding, and activating vaccine-reactive T cells in the tumor microenvironment (TME) and by opposing immunosuppressive mechanisms such as the presence of regulatory T cells and upregulation of immune checkpoints. Here, we discuss immuno-oncology agents that could potentially enhance the immune responses generated by ERV-targeting vaccines against ERV-expressing tumors (Fig. 4).

### Checkpoint inhibitors

One of the mechanisms by which tumors avoid immune surveillance is by upregulating inhibitory pathways such as the PD-1/PD-L1 axis. PD-1 and CTLA-4 are immune checkpoints expressed on T cells that, following binding to their cognate ligands, hinder the cytotoxic function of T cells (111–113). Previous studies have demonstrated that ICIs, such as those targeting PD-1/PD-L1 or CTL4, can expand the amount of neoepitope-specific CD8^+^ T cells infiltrating the TME and decrease the negative regulation of T-cell activation. Additional studies have demonstrated enhanced tumor regression, prolonged survival, increased cytotoxic T-cell responses, and protection from tumor rechallenge following combination with ICIs (114, 115).

As shown in Table 4, so far, most of the ERV-targeting vaccines studied preclinically have been tested in combination with anti–PD-1 (104–106). The addition of PD-1 blockade has generally enhanced the antitumor immunity of ERV vaccines, with some reports of tumor eradication following treatment (105, 109). Therefore, the combination of ERV-targeting vaccines plus ICIs continues to be a promising strategy, the efficacy of which should further be evaluated in clinical studies.

### Cytokines

Therapeutic ERV cancer vaccines may offer a promising avenue to accomplish tumor regression in tumors with a high ERV expression; however, they may offer limited clinical benefit when other mechanisms of immune failure are also at play. Cytokines and chemokines within the TME regulate immune responses and cellular processes such as proliferation, apoptosis, and differentiation, all of which impact the efficacy of cancer therapies including immunotherapy (116). Some of the cytokines with reported protumor activity include TGF-β, IL-6, CXCL8, VEGF, and colony stimulating factor-1. Strategies for neutralizing these cytokines within the TME utilizing either antibodies or small-molecule inhibitors are currently being evaluated clinically. On the other hand, cytokines such as IFNγ, GM-CSF, IL-2, IL-12, and IL-15 have demonstrated antitumor effects in preclinical studies (117). These cytokines slow growth by inhibiting tumor cell proliferation and stimulating an antitumor immune response. Hence, a therapeutic strategy that could potentiate the immune responses of ERV-targeting vaccines in tumors could either block the production/function of immunosuppressive cytokines/chemokines or administer immunostimulatory cytokines, such as NHS–IL-12 or N803, as combination therapy targeting ERV proteins (118, 119).

NHS–IL-12 is a fusion protein consisting of IL-12 bound to an antibody that targets DNA in necrotic areas present in solid tumors (119). Necrosis supports the targeted delivery of IL-12 to the tumor and exposes intracellular antigens within the TME that could further be targeted with cancer vaccines. Mice treated with NHS–IL-2 have been found to have long-lasting lytic CD8^+^ T-cell responses specific to the endogenous retroviral protein p15E (120). It has been reported that the combination with NHS–IL-12 allows for vaccine-induced T cells to better infiltrate the tumor and remain active (120–122).

On the other hand, N803 (Anktiva) is an IL-15 superagonist recently approved by the FDA that binds to circulating immune cells through the IL-15 receptor, leading to the activation and expansion of NK cells and central memory T cells (118, 120, 123–125). Treatment of mice with a combination of N803 and therapeutic neoepitope cancer vaccine also promoted the expansion of p15E-specific T cells in the MC38 colorectal tumor model (120). The addition of NHS–IL-12 and anti–PD-L1 to the regimen further enhanced tumor regression and increased CD8^+^ T-cell infiltration and clonality of T cells in the TME (120).

Altogether, combining these immuno-oncology agents with therapeutic ERV cancer vaccines could potentially increase the immunogenicity of the vaccine to levels capable of rejecting the tumor. Although the combination of cytokines with ERV vaccines has yet to be evaluated preclinically, there is the potential to obtain enhanced antitumor immune responses based on previous studies evaluating the combined regimen of therapeutic cancer vaccines and cytokines at the preclinical and clinical levels.

### Targeting epigenetic regulation of ERV sequences

It is well known that ERV expression is regulated epigenetically. Among the epigenetic processes that are relevant for controlling ERV expression are CpG methylation, histone deacetylation, and histone methylation. CpG-rich promoters tend to indicate the silencing of corresponding genes through methylation. There is evidence that hypomethylation of the genome leads to expression of ERVs in cancerous tissues (41). It has been reported that the use of DNA methyl transferase inhibitors (DNMTi), such as azacitidine (Aza), enhances the expression of specific transcripts of ERVs in several cancer types, such as ovarian cancer, endometrial cancer, melanoma, and neuroblastoma, by removing methylation from promoter regions of silenced ERVs (41, 79).

Prior studies have also demonstrated that DNMTis can induce “viral mimicry” by producing ERV transcripts that could activate innate immunity and stimulate viral defense response mechanisms (126). Chiappinelli and colleagues (79) show that treatment with Aza upregulates sense and antisense ERV transcripts, and that this bidirectional transcription results in double-stranded RNA (dsRNA) transcripts that trigger type I IFN responses and apoptosis in ovarian cancer cell lines. Furthermore, DNMTis can also promote the expression of antitumor cytokines such as IL-2, IFNγ, and Th1 chemokines like CXCL9, which could then enhance the infiltration of effector T cells to the TME (127). Hence, combination treatment with DNMTis may sensitize the response to ERV cancer vaccines by further increasing tumor cell visibility to immune surveillance, intensifying the antigen presentation process, enhancing the trafficking of effector T cells, and promoting cytolytic T-cell responses in the TME (128).

Another epigenetic mechanism that could regulate the expression of ERVs is histone deacetylation. Acetylation of lysine residues in histones is catalyzed by histone acetyltransferases and counteracted by histone deacetylases (HDAC). Nonetheless, previous studies have reported that the use of HDAC inhibitors (HDACi) as a monotherapy have failed to significantly increase ERV expression in humans (129). However, there is a possibility that histone deacetylation may act in combination with CpG methylation to regulate ERV expression; therefore, additional studies are needed to further understand this mechanism.

In addition to modulating ERV expression, immunogenic neoantigens derived from ERV elements can also be induced by DNMTis and HDACis (130). Targeting neoantigens represents a way to increase antitumor immune responses (131). Goyal and colleagues (130) recently identified 45 validated ERV-derived neoantigens that were presented by HLAs upon treatment with DNMTis and HDACis. This phenomenon was conserved across lung cancer, colon cancer, glioblastoma, and AML cell lines *in vitro* and in patients with cancer as the authors identified ERV-derived neoepitopes in patients with AML who were treated with the hypomethylating agent decitabine. More importantly, these neoantigens were able to activate T cells and elicit cytotoxic responses against tumor cells. This immune recognition could potentially be enhanced by addition of autophagy inhibitors as a combination strategy, which could inhibit the degradation of endogenous retroelement transcripts induced by Aza, increasing their capacity to generate MHC-I–associated peptides, as shown in a recent study in AML (132).

## Conclusions

The aberrant expression of endogenous retroviral proteins within human carcinomas represents a class of tumor-associated antigens that can potentially be immunologically targeted in cancer. There are currently several diverse treatment modalities being evaluated in preclinical models to target ERVs, including the use of ERV-specific antibodies, ERV-specific T-cell transfer, and ERV-targeting therapeutic cancer vaccines. Combinations with other immuno-oncology agents such as ICIs, cytokines, and epigenetic regulators could further enhance antitumor immune responses by reducing immunosuppression in the TME, augmenting the antigen presentation process, promoting cytolytic T-cell responses, and increasing tumor cell visibility to immune surveillance. So far, most studies conducted are still in an early stage, with preclinical studies demonstrating preliminary antitumor efficacy in various tumor models. Nonetheless, careful selection of the target ERV is key to ensure minimal or no expression in normal tissues and reduce off-target effects during treatment. Altogether, mounting preclinical evidence strongly supports the continued development of ERV-targeting strategies as a potential treatment for tumors with high ERV expression.

## References

[1] Bannert N , Kurth R. The evolutionary dynamics of human endogenous retroviral families. Annu Rev Genomics Hum Genet 2006;7:149–73. 16722807 10.1146/annurev.genom.7.080505.115700

[2] Song Y , Li X, Wei X, Cui J. Human endogenous retroviruses as biomedicine markers. Virol Sin 2021;36:852–8. 33905075 10.1007/s12250-021-00387-7 PMC8558139

[3] Stocking C , Kozak CA. Murine endogenous retroviruses. Cell Mol Life Sci 2008;65:3383–98. 18818872 10.1007/s00018-008-8497-0 PMC4802364

[4] Vargiu L , Rodriguez-Tomé P, Sperber GO, Cadeddu M, Grandi N, Blikstad V, . Classification and characterization of human endogenous retroviruses; mosaic forms are common. Retrovirology 2016;13:7. 26800882 10.1186/s12977-015-0232-y PMC4724089

[5] Coffin JM . Structure and classification of retroviruses. In: Levy JA, editor. The Retroviridae. Boston, MA: Springer US; 1992. p. 19–49.

[6] de Parseval N , Heidmann T. Human endogenous retroviruses: from infectious elements to human genes. Cytogenet Genome Res 2005;110:318–32. 16093684 10.1159/000084964

[7] Feschotte C , Gilbert C. Endogenous viruses: insights into viral evolution and impact on host biology. Nat Rev Genet 2012;13:283–96. 22421730 10.1038/nrg3199

[8] Hurst TP , Magiorkinis G. Epigenetic control of human endogenous retrovirus expression: focus on regulation of long-terminal repeats (LTRs). Viruses 2017;9:130. 28561791 10.3390/v9060130 PMC5490807

[9] Ehrlich M . DNA hypomethylation in cancer cells. Epigenomics 2009;1:239–59. 20495664 10.2217/epi.09.33 PMC2873040

[10] Müller MD , Holst PJ, Nielsen KN. A systematic review of expression and immunogenicity of human endogenous retroviral proteins in cancer and discussion of therapeutic approaches. Int J Mol Sci 2022;23:1330. 35163254 10.3390/ijms23031330 PMC8836156

[11] Wang-Johanning F , Radvanyi L, Rycaj K, Plummer JB, Yan P, Sastry KJ, . Human endogenous retrovirus K triggers an antigen-specific immune response in breast cancer patients. Cancer Res 2008;68:5869–77. 18632641 10.1158/0008-5472.CAN-07-6838 PMC5802396

[12] Rycaj K , Plummer JB, Yin B, Li M, Garza J, Radvanyi L, . Cytotoxicity of human endogenous retrovirus K–specific T cells toward autologous ovarian cancer cells. Clin Cancer Res 2015;21:471–83. 25370465 10.1158/1078-0432.CCR-14-0388

[13] Cherkasova E , Scrivani C, Doh S, Weisman Q, Takahashi Y, Harashima N, . Detection of an immunogenic HERV-E envelope with selective expression in clear cell kidney cancer. Cancer Res 2016;76:2177–85. 26862115 10.1158/0008-5472.CAN-15-3139 PMC4873424

[14] Hahn S , Ugurel S, Hanschmann K-M, Strobel H, Tondera C, Schadendorf D, . Serological response to human endogenous retrovirus K in melanoma patients correlates with survival probability. AIDS Res Hum Retroviruses 2008;24:717–23. 18462078 10.1089/aid.2007.0286

[15] Wang-Johanning F , Li M, Esteva FJ, Hess KR, Yin B, Rycaj K, . Human endogenous retrovirus type K antibodies and mRNA as serum biomarkers of early-stage breast cancer. Int J Cancer 2014;134:587–95. 23873154 10.1002/ijc.28389 PMC4191919

[16] Gimenez J , Montgiraud C, Pichon J-P, Bonnaud B, Arsac M, Ruel K, . Custom human endogenous retroviruses dedicated microarray identifies self-induced HERV-W family elements reactivated in testicular cancer upon methylation control. Nucleic Acids Res 2010;38:2229–46. 20053729 10.1093/nar/gkp1214 PMC2853125

[17] Yu J , Qiu P, Ai J, Liu B, Han G-Z, Zhu F, . Endogenous retrovirus activation: potential for immunology and clinical applications. Natl Sci Rev 2024;11:nwae034. 38495812 10.1093/nsr/nwae034 PMC10941811

[18] Johnson WE . Origins and evolutionary consequences of ancient endogenous retroviruses. Nat Rev Microbiol 2019;17:355–70. 30962577 10.1038/s41579-019-0189-2

[19] Rowe HM , Trono D. Dynamic control of endogenous retroviruses during development. Virology 2011;411:273–87. 21251689 10.1016/j.virol.2010.12.007

[20] Fu B , Ma H, Liu D. Endogenous retroviruses function as gene expression regulatory elements during mammalian pre-implantation embryo development. Int J Mol Sci 2019;20:790. 30759824 10.3390/ijms20030790 PMC6387303

[21] Liu X , Liu Z, Wu Z, Ren J, Fan Y, Sun L, . Resurrection of endogenous retroviruses during aging reinforces senescence. Cell 2023;186:287–304.e26. 36610399 10.1016/j.cell.2022.12.017

[22] Buttler CA , Chuong EB. Emerging roles for endogenous retroviruses in immune epigenetic regulation. Immunol Rev 2022;305:165–78. 34816452 10.1111/imr.13042 PMC8766910

[23] Russ E , Iordanskiy S. Endogenous retroviruses as modulators of innate immunity. Pathogens 2023;12:162. 36839434 10.3390/pathogens12020162 PMC9963469

[24] Chuong EB . The placenta goes viral: retroviruses control gene expression in pregnancy. PLoS Biol 2018;16:e3000028. 30300353 10.1371/journal.pbio.3000028 PMC6177113

[25] Mi S , Lee X, Li X, Veldman GM, Finnerty H, Racie L, . Syncytin is a captive retroviral envelope protein involved in human placental morphogenesis. Nature 2000;403:785–9. 10693809 10.1038/35001608

[26] Wang J , Lu X, Zhang W, Liu G-H. Endogenous retroviruses in development and health. Trends Microbiol 2024;32:342–54. 37802660 10.1016/j.tim.2023.09.006

[27] Dopkins N , Nixon DF. Activation of human endogenous retroviruses and its physiological consequences. Nat Rev Mol Cell Biol 2024;25:212–22. 37872387 10.1038/s41580-023-00674-z

[28] Xiang Y , Liang H. The regulation and functions of endogenous retrovirus in embryo development and stem cell differentiation. Stem Cell Int 2021;2021:6660936. 10.1155/2021/6660936 PMC7937486 33727936

[29] Göke J , Lu X, Chan Y-S, Ng H-H, Ly L-H, Sachs F, . Dynamic transcription of distinct classes of endogenous retroviral elements marks specific populations of early human embryonic cells. Cell Stem Cell 2015;16:135–41. 25658370 10.1016/j.stem.2015.01.005

[30] Frank JA , Singh M, Cullen HB, Kirou RA, Benkaddour-Boumzaouad M, Cortes JL, . Evolution and antiviral activity of a human protein of retroviral origin. Science 2022;378:422–8. 36302021 10.1126/science.abq7871 PMC10542854

[31] Malfavon-Borja R , Feschotte C. Fighting fire with fire: endogenous retrovirus envelopes as restriction factors. J Virol 2015;89:4047–50. 25653437 10.1128/JVI.03653-14 PMC4442362

[32] Kurth R , Bannert N. Beneficial and detrimental effects of human endogenous retroviruses. Int J Cancer 2010;126:306–14. 19795446 10.1002/ijc.24902

[33] Szpakowski S , Sun X, Lage JM, Dyer A, Rubinstein J, Kowalski D, . Loss of epigenetic silencing in tumors preferentially affects primate-specific retroelements. Gene 2009;448:151–67. 19699787 10.1016/j.gene.2009.08.006 PMC2783545

[34] Kato M , Takemoto K, Shinkai Y. A somatic role for the histone methyltransferase Setdb1 in endogenous retrovirus silencing. Nat Commun 2018;9:1683. 29703894 10.1038/s41467-018-04132-9 PMC5923290

[35] Wolf D , Goff SP. Embryonic stem cells use ZFP809 to silence retroviral DNAs. Nature 2009;458:1201–4. 19270682 10.1038/nature07844 PMC2676211

[36] Schultz DC , Ayyanathan K, Negorev D, Maul GG, Rauscher FJ III. SETDB1: a novel KAP-1-associated histone H3, lysine 9-specific methyltransferase that contributes to HP1-mediated silencing of euchromatic genes by KRAB zinc-finger proteins. Genes Dev 2002;16:919–32. 11959841 10.1101/gad.973302 PMC152359

[37] McGeary MK , Damsky W, Daniels AJ, Lang SM, Xu Q, Song E, . Setdb1 loss induces type I interferons and immune clearance of melanoma. Cancer Immunol Res 2025;13:245–57. 39589394 10.1158/2326-6066.CIR-23-0514

[38] Pan D , Bao X, Hu M, Jiao M, Li F, Li CY. SETDB1 restrains endogenous retrovirus expression and antitumor immunity during radiotherapy. Cancer Res 2022;82:2748–60. 35648422 10.1158/0008-5472.CAN-21-3523 PMC9357127

[39] Lin J , Guo D, Liu H, Zhou W, Wang C, Müller I, . The SETDB1-TRIM28 complex suppresses antitumor immunity. Cancer Immunol Res 2021;9:1413–24. 34848497 10.1158/2326-6066.CIR-21-0754 PMC8647838

[40] Alcazer V , Bonaventura P, Tonon L, Michel E, Mutez V, Fabres C, . HERVs characterize normal and leukemia stem cells and represent a source of shared epitopes for cancer immunotherapy. Am J Hematol 2022;97:1200–14. 35759575 10.1002/ajh.26647 PMC9540360

[41] Gao Y , Yu X-F, Chen T. Human endogenous retroviruses in cancer: expression, regulation and function. Oncol Lett 2021;21:121. 33552242 10.3892/ol.2020.12382 PMC7798031

[42] Vergara Bermejo A , Ragonnaud E, Daradoumis J, Holst P. Cancer associated endogenous retroviruses: ideal immune targets for adenovirus-based immunotherapy. Int J Mol Sci 2020;21:4843. 32650622 10.3390/ijms21144843 PMC7402293

[43] Stricker E , Peckham-Gregory EC, Scheurer ME. CancerHERVdb: human endogenous retrovirus (HERV) expression database for human cancer accelerates studies of the retrovirome and predictions for HERV-based therapies. J Virol 2023;97:e0005923. 37255431 10.1128/jvi.00059-23 PMC10308937

[44] Larsen JM , Christensen IJ, Nielsen HJ, Hansen U, Bjerregaard B, Talts JF, . Syncytin immunoreactivity in colorectal cancer: potential prognostic impact. Cancer Lett 2009;280:44–9. 19327884 10.1016/j.canlet.2009.02.008

[45] Díaz-Carballo D , Acikelli AH, Klein J, Jastrow H, Dammann P, Wyganowski T, . Therapeutic potential of antiviral drugs targeting chemorefractory colorectal adenocarcinoma cells overexpressing endogenous retroviral elements. J Exp Clin Cancer Res 2015;34:81. 26260344 10.1186/s13046-015-0199-5 PMC4542094

[46] Zhou Y , Liu L, Liu Y, Zhou P, Yan Q, Yu H, . Implication of human endogenous retrovirus W family envelope in hepatocellular carcinoma promotes MEK/ERK-mediated metastatic invasiveness and doxorubicin resistance. Cell Death Discov 2021;7:177. 34238921 10.1038/s41420-021-00562-5 PMC8266889

[47] Yu H , Liu T, Zhao Z, Chen Y, Zeng J, Liu S, . Mutations in 3′-long terminal repeat of HERV-W family in chromosome 7 upregulate syncytin-1 expression in urothelial cell carcinoma of the bladder through interacting with c-Myb. Oncogene 2014;33:3947–58. 24013223 10.1038/onc.2013.366

[48] Yan H , Qiu W, Koehne de Gonzalez AK, Wei J-S, Tu M, Xi C-H, . HHLA2 is a novel immune checkpoint protein in pancreatic ductal adenocarcinoma and predicts post-surgical survival. Cancer Lett 2019;442:333–40. 30447255 10.1016/j.canlet.2018.11.007 PMC6357962

[49] Xiao Y , Li H, Yang L-L, Mao L, Wu C-C, Zhang W-F, . The expression patterns and associated clinical parameters of human endogenous retrovirus-H long terminal repeat-associating protein 2 and transmembrane and immunoglobulin domain containing 2 in oral squamous cell carcinoma. Dis Markers 2019;2019:5421985. 31089395 10.1155/2019/5421985 PMC6476002

[50] Zhu Z , Dong W. Overexpression of HHLA2, a member of the B7 family, is associated with worse survival in human colorectal carcinoma. Onco Targets Ther 2018;11:1563–70. 29593422 10.2147/OTT.S160493 PMC5865557

[51] Jing C-Y , Fu Y-P, Yi Y, Zhang M-X, Zheng S-S, Huang J-L, . HHLA2 in intrahepatic cholangiocarcinoma: an immune checkpoint with prognostic significance and wider expression compared with PD-L1. J Immunother Cancer 2019;7:77. 30885276 10.1186/s40425-019-0554-8 PMC6421676

[52] Luo M , Lin Y, Liang R, Li Y, Ge L. Clinical significance of the HHLA2 protein in hepatocellular carcinoma and the tumor microenvironment. J Inflamm Res 2021;14:4217–28. 34483677 10.2147/JIR.S324336 PMC8409601

[53] Wang R , Guo H, Tang X, Zhang T, Liu Y, Zhang C, . Interferon gamma-induced interferon regulatory factor 1 activates transcription of HHLA2 and induces immune escape of hepatocellular carcinoma cells. Inflammation 2022;45:308–30. 34536158 10.1007/s10753-021-01547-3

[54] Farrag MS , Ibrahim EM, El-Hadidy TA, Akl MF, Elsergany AR, Abdelwahab HW. Human endogenous retrovirus-H long terminal repeat-associating protein 2 (HHLA2) is a novel immune checkpoint protein in lung cancer which predicts survival. Asian Pac J Cancer Prev 2021;22:1883–9. 34181347 10.31557/APJCP.2021.22.6.1883 PMC8418860

[55] Chen L , Zhu D, Feng J, Zhou Y, Wang Q, Feng H, . Overexpression of HHLA2 in human clear cell renal cell carcinoma is significantly associated with poor survival of the patients. Cancer Cell Int 2019;19:101. 31015801 10.1186/s12935-019-0813-2 PMC6469208

[56] Van Tongelen A , Loriot A, De Smet C. Oncogenic roles of DNA hypomethylation through the activation of cancer-germline genes. Cancer Lett 2017;396:130–7. 28342986 10.1016/j.canlet.2017.03.029

[57] Chen B , Xu K, Zhang Y, Xu P, Li C, Liu J, . LncRNA ERVH48-1 contributes to the Drug resistance of prostate cancer and proliferation through sponging of miR-4784 to the activation of the wnt/β-catenin pathway. Cancers (Basel) 2023;15:1902. 36980789 10.3390/cancers15061902 PMC10046998

[58] Záveský L , Jandáková E, Weinberger V, Minář L, Kohoutová M, Slanař O. Human endogenous retroviruses (HERVs) in breast cancer: altered expression pattern implicates divergent roles in carcinogenesis. Oncology 2024;102:858–67. 38408442 10.1159/000538021 PMC11449185

[59] Shigemura T , Perrot N, Huang Z, Bhatt RS, Sheshdeh AB, Ahmar NE, . Regulation of HHLA2 expression in kidney cancer and myeloid cells. BMC Cancer 2023;23:1039. 37891555 10.1186/s12885-023-11496-9 PMC10605970

[60] Wei Y , Ren X, Galbo PM Jr, Moerdler S, Wang H, Sica RA, . KIR3DL3-HHLA2 is a human immunosuppressive pathway and a therapeutic target. Sci Immunol 2021;6:eabf9792. 34244312 10.1126/sciimmunol.abf9792 PMC9744578

[61] Au L , Hatipoglu E, Robert de Massy M, Litchfield K, Beattie G, Rowan A, . Determinants of anti-PD-1 response and resistance in clear cell renal cell carcinoma. Cancer Cell 2021;39:1497–518.e11. 34715028 10.1016/j.ccell.2021.10.001 PMC8599450

[62] Ng KW , Boumelha J, Enfield KSS, Almagro J, Cha H, Pich O, . Antibodies against endogenous retroviruses promote lung cancer immunotherapy. Nature 2023;616:563–73. 37046094 10.1038/s41586-023-05771-9 PMC10115647

[63] Lecuelle J , Favier L, Fraisse C, Lagrange A, Kaderbhai C, Boidot R, . MER4 endogenous retrovirus correlated with better efficacy of anti-PD1/PD-L1 therapy in non-small cell lung cancer. J ImmunoTherapy Cancer 2022;10:e004241. 10.1136/jitc-2021-004241 PMC8919440 35277462

[64] Panda A , de Cubas AA, Stein M, Riedlinger G, Kra J, Mayer T, . Endogenous retrovirus expression is associated with response to immune checkpoint blockade in clear cell renal cell carcinoma. JCI Insight 2018;3:e121522. 30135306 10.1172/jci.insight.121522 PMC6141170

[65] Zhou J-G , Zeng Y, Wang H, Jin S-H, Wang Y-J, He S, . Identification of an endogenous retroviral signature to predict anti-PD1 response in advanced clear cell renal cell carcinoma: an integrated analysis of three clinical trials. Ther Adv Med Oncol 2022;14:17588359221126154. 37614979 10.1177/17588359221126154 PMC10442641

[66] Topham JT , Titmuss E, Pleasance ED, Williamson LM, Karasinska JM, Culibrk L, . Endogenous retrovirus transcript levels are associated with immunogenic signatures in multiple metastatic cancer types. Mol Cancer Ther 2020;19:1889–97. 32518206 10.1158/1535-7163.MCT-20-0094

[67] Rasmussen KD , Helin K. Role of TET enzymes in DNA methylation, development, and cancer. Genes Dev 2016;30:733–50. 27036965 10.1101/gad.276568.115 PMC4826392

[68] Kassiotis G . Endogenous retroviruses and the development of cancer. J Immunol 2014;192:1343–9. 24511094 10.4049/jimmunol.1302972 PMC3925786

[69] Shah NM , Jang HJ, Liang Y, Maeng JH, Tzeng S-C, Wu A, . Pan-cancer analysis identifies tumor-specific antigens derived from transposable elements. Nat Genet 2023;55:631–9. 36973455 10.1038/s41588-023-01349-3 PMC13152471

[70] Merlotti A , Sadacca B, Arribas YA, Ngoma M, Burbage M, Goudot C, . Noncanonical splicing junctions between exons and transposable elements represent a source of immunogenic recurrent neo-antigens in patients with lung cancer. Sci Immunol 2023;8:eabm6359. 36735774 10.1126/sciimmunol.abm6359

[71] Bonaventura P , Alcazer V, Mutez V, Tonon L, Martin J, Chuvin N, . Identification of shared tumor epitopes from endogenous retroviruses inducing high-avidity cytotoxic T cells for cancer immunotherapy. Sci Adv 2022;8:eabj3671. 35080970 10.1126/sciadv.abj3671 PMC8791462

[72] Kobayashi S , Tokita S, Moniwa K, Kitahara K, Iuchi H, Matsuo K, . Proteogenomic identification of an immunogenic antigen derived from human endogenous retrovirus in renal cell carcinoma. JCI Insight 2023;8:e167712. 37606040 10.1172/jci.insight.167712 PMC10543709

[73] Rakoff-Nahoum S , Kuebler PJ, Heymann JJ, E Sheehy M, Ortiz GM, S Ogg G, . Detection of T Lymphocytes specific for human endogenous retrovirus K (HERV-K) in patients with seminoma. AIDS Res Hum Retroviruses 2006;22:52–6. 16438646 10.1089/aid.2006.22.52

[74] Schiavetti F , Thonnard J, Colau D, Boon T, Coulie PG. A human endogenous retroviral sequence encoding an antigen recognized on melanoma by cytolytic T lymphocytes. Cancer Res 2002;62:5510–6. 12359761

[75] Takahashi Y , Harashima N, Kajigaya S, Yokoyama H, Cherkasova E, McCoy JP, . Regression of human kidney cancer following allogeneic stem cell transplantation is associated with recognition of an HERV-E antigen by T cells. J Clin Invest 2008;118:1099–109. 18292810 10.1172/JCI34409 PMC2248804

[76] Sauter M , Schommer S, Kremmer E, Remberger K, Dölken G, Lemm I, . Human endogenous retrovirus K10: expression of Gag protein and detection of antibodies in patients with seminomas. J Virol 1995;69:414–21. 7983737 10.1128/jvi.69.1.414-421.1995 PMC188589

[77] Kleiman A , Senyuta N, Tryakin A, Sauter M, Karseladze A, Tjulandin S, . HERV-K(HML-2) GAG/ENV antibodies as indicator for therapy effect in patients with germ cell tumors. Int J Cancer 2004;110:459–61. 15095315 10.1002/ijc.11649

[78] Attermann AS , Bjerregaard A-M, Saini SK, Grønbæk K, Hadrup SR. Human endogenous retroviruses and their implication for immunotherapeutics of cancer. Ann Oncol 2018;29:2183–91. 30239576 10.1093/annonc/mdy413

[79] Chiappinelli KB , Strissel PL, Desrichard A, Li H, Henke C, Akman B, . Inhibiting DNA methylation causes an interferon response in cancer via dsRNA including endogenous retroviruses. Cell 2015;162:974–86. 26317466 10.1016/j.cell.2015.07.011 PMC4556003

[80] Saini SK , Ørskov AD, Bjerregaard A-M, Unnikrishnan A, Holmberg-Thydén S, Borch A, . Human endogenous retroviruses form a reservoir of T cell targets in hematological cancers. Nat Commun 2020;11:5660. 33168830 10.1038/s41467-020-19464-8 PMC7653045

[81] Krishnamurthy J , Rabinovich BA, Mi T, Switzer KC, Olivares S, Maiti SN, . Genetic engineering of T cells to target HERV-K, an ancient retrovirus on melanoma. Clin Cancer Res 2015;21:3241–51. 25829402 10.1158/1078-0432.CCR-14-3197 PMC4506228

[82] Mullins CS , Linnebacher M. Endogenous retrovirus sequences as a novel class of tumor-specific antigens: an example of HERV-H env encoding strong CTL epitopes. Cancer Immunol Immunother 2012;61:1093–100. 22187063 10.1007/s00262-011-1183-3 PMC11029769

[83] Denner J . Immunosuppression by retroviruses: implications for xenotransplantation. Ann N Y Acad Sci 1998;862:75–86. 9928208 10.1111/j.1749-6632.1998.tb09119.x

[84] Kraus B , Fischer K, Büchner S, Sliva K, Schnierle B. Vaccination directed against the human endogenous retrovirus-K envelope protein shows efficiency in a murine tumor model system. Retrovirology 2013;10:P81. 10.1371/journal.pone.0072756 PMC3758348 24023643

[85] Oostendorp RA , Meijer CJ, Scheper RJ. Immunosuppression by retroviral-envelope-related proteins, and their role in non-retroviral human disease. Crit Rev Oncol Hematol 1993;14:189–206. 8397847 10.1016/1040-8428(93)90009-s

[86] Cianciolo GJ , Copeland TD, Oroszlan S, Snyderman R. Inhibition of lymphocyte proliferation by a synthetic peptide homologous to retroviral envelope proteins. Science 1985;230:453–5. 2996136 10.1126/science.2996136

[87] Mangeney M , de Parseval N, Thomas G, Heidmann T. The full-length envelope of an HERV-H human endogenous retrovirus has immunosuppressive properties. J Gen Virol 2001;82:2515–8. 11562544 10.1099/0022-1317-82-10-2515

[88] Fuchs NV , Loewer S, Daley GQ, Izsvák Z, Löwer J, Löwer R. Human endogenous retrovirus K (HML-2) RNA and protein expression is a marker for human embryonic and induced pluripotent stem cells. Retrovirology 2013;10:115. 24156636 10.1186/1742-4690-10-115 PMC3819666

[89] Kämmerer U , Germeyer A, Stengel S, Kapp M, Denner J. Human endogenous retrovirus K (HERV-K) is expressed in villous and extravillous cytotrophoblast cells of the human placenta. J Reprod Immunol 2011;91:1–8. 21840605 10.1016/j.jri.2011.06.102

[90] Bergallo M , Marozio L, Botta G, Tancredi A, Daprà V, Galliano I, . Human endogenous retroviruses are preferentially expressed in mononuclear cells from cord blood than from maternal blood and in the fetal part of placenta. Front Pediatr 2020;8:244. 32478020 10.3389/fped.2020.00244 PMC7240011

[91] Holder BS , Tower CL, Forbes K, Mulla MJ, Aplin JD, Abrahams VM. Immune cell activation by trophoblast-derived microvesicles is mediated by syncytin 1. Immunology 2012;136:184–91. 22348442 10.1111/j.1365-2567.2012.03568.x PMC3403269

[92] Heidmann O , Béguin A, Paternina J, Berthier R, Deloger M, Bawa O, . HEMO, an ancestral endogenous retroviral envelope protein shed in the blood of pregnant women and expressed in pluripotent stem cells and tumors. Proc Natl Acad Sci U S A 2017;114:E6642–51. 28739914 10.1073/pnas.1702204114 PMC5559007

[93] Burn A , Roy F, Freeman M, Coffin JM. Widespread expression of the ancient HERV-K (HML-2) provirus group in normal human tissues. PLoS Biol 2022;20:e3001826. 36256614 10.1371/journal.pbio.3001826 PMC9578601

[94] Schmitt K , Heyne K, Roemer K, Meese E, Mayer J. HERV-K(HML-2) rec and np9 transcripts not restricted to disease but present in many normal human tissues. Mobile DNA 2015;6:4. 25750667 10.1186/s13100-015-0035-7 PMC4351823

[95] Sacha JB , Kim I-J, Chen L, Ullah JH, Goodwin DA, Simmons HA, . Vaccination with cancer- and HIV infection-associated endogenous retrotransposable elements is safe and immunogenic. J Immunol 2012;189:1467–79. 22745376 10.4049/jimmunol.1200079 PMC3401319

[96] Herve CA , Lugli EB, Brand A, Griffiths DJ, Venables PJW. Autoantibodies to human endogenous retrovirus-K are frequently detected in health and disease and react with multiple epitopes. Clin Exp Immunol 2002;128:75–82. 11982593 10.1046/j.1365-2249.2002.01735.x PMC1906363

[97] Chisca M , Larouche J-D, Xing Q, Kassiotis G. Antibodies against endogenous retroviruses. Immunol Rev 2024;328:300–13. 39152687 10.1111/imr.13378 PMC11659944

[98] Nadal R , Barisic S, Scurti GM, Cherkasova E, Chen L, Wood K, . Final results of a phase I trial of HERV-E TCR transduced T cells for the treatment of HLA-A*11 patients with metastatic clear cell renal cell carcinoma (mccRCC). J Clin Oncol 2024;42(Suppl 4):435.

[99] Wang-Johanning F , Rycaj K, Plummer JB, Li M, Yin B, Frerich K, . Immunotherapeutic potential of anti-human endogenous retrovirus-K envelope protein antibodies in targeting breast tumors. J Natl Cancer Inst 2012;104:189–210. 22247020 10.1093/jnci/djr540 PMC3274512

[100] Lang MS , Oostendorp RA, Simons PJ, Boersma W, Knegt P, van Ewijk W. New monoclonal antibodies against the putative immunosuppressive site of retroviral p15E. Cancer Res 1994;54:1831–6. 7511054

[101] Zhou F , Krishnamurthy J, Wei Y, Li M, Hunt K, Johanning GL, . Chimeric antigen receptor T cells targeting HERV-K inhibit breast cancer and its metastasis through downregulation of Ras. OncoImmunology 2015;4:e1047582. 26451325 10.1080/2162402X.2015.1047582 PMC4589998

[102] Nadal R , Barisic S, Scurti GM, Cherkasova E, Chen L, Wood K, . Phase I results of human endogenous retrovirus type-E (HERV-E) TCR transduced T-cells in patients (pts) with metastatic clear cell renal cell carcinoma (mccRCC). J Clin Oncol 2023;41(Suppl 16):2549.

[103] Kraus B , Fischer K, Sliva K, Schnierle BS. Vaccination directed against the human endogenous retrovirus-K (HERV-K) gag protein slows HERV-K gag expressing cell growth in a murine model system. Virol J 2014;11:58. 24669861 10.1186/1743-422X-11-58 PMC3974434

[104] Neukirch L , Nielsen TK, Laursen H, Daradoumis J, Thirion C, Holst PJ. Adenovirus based virus-like-vaccines targeting endogenous retroviruses can eliminate growing colorectal cancers in mice. Oncotarget 2019;10:1458–72. 30858929 10.18632/oncotarget.26680 PMC6402721

[105] Daradoumis J , Ragonnaud E, Skandorff I, Nielsen KN, Bermejo AV, Andersson A-M, . An endogenous retrovirus vaccine encoding an envelope with a mutated immunosuppressive domain in combination with anti-PD1 treatment eradicates established tumours in mice. Viruses 2023;15:926. 37112906 10.3390/v15040926 PMC10141008

[106] Peltonen K , Feola S, Umer HM, Chiaro J, Mermelekas G, Ylösmäki E, . Therapeutic cancer vaccination with immunopeptidomics-discovered antigens confers protective antitumor efficacy. Cancers (Basel) 2021;13:3408. 34298622 10.3390/cancers13143408 PMC8306067

[107] Skandorff I , Ragonnaud E, Gille J, Andersson A-M, Schrödel S, Duvnjak L, . Human Ad19a/64 HERV-W vaccines uncover immunosuppression domain-dependent T-cell response differences in inbred mice. Int J Mol Sci 2023;24:9972. 37373123 10.3390/ijms24129972 PMC10297909

[108] Scrimieri F , Askew D, Corn DJ, Eid S, Bobanga ID, Bjelac JA, . Murine leukemia virus envelope gp70 is a shared biomarker for the high-sensitivity quantification of murine tumor burden. Oncoimmunology 2013;2:e26889. 24482753 10.4161/onci.26889 PMC3894233

[109] Maldonado MDM , Iida M, Hernandez MDMG, Le L, Donahue RN, Palena C, . Abstract 4092: characterizing the endogenous retroviral envelope protein ERVMER34-1 as a target for a therapeutic cancer vaccine. Cancer Res 2024;84(Suppl 6):4092.

[110] Mangeney M , Renard M, Schlecht-Louf G, Bouallaga I, Heidmann O, Letzelter C, . Placental syncytins: genetic disjunction between the fusogenic and immunosuppressive activity of retroviral envelope proteins. Proc Natl Acad Sci U S A 2007;104:20534–9. 18077339 10.1073/pnas.0707873105 PMC2154466

[111] Santiago-Sánchez GS , Hodge JW, Fabian KP. Tipping the scales: immunotherapeutic strategies that disrupt immunosuppression and promote immune activation. Front Immunol 2022;13:993624. 36159809 10.3389/fimmu.2022.993624 PMC9492957

[112] He X , Xu C. Immune checkpoint signaling and cancer immunotherapy. Cell Res 2020;30:660–9. 32467592 10.1038/s41422-020-0343-4 PMC7395714

[113] Mejía-Guarnizo LV , Monroy-Camacho PS, Turizo-Smith AD, Rodríguez-García JA. The role of immune checkpoints in antitumor response: a potential antitumor immunotherapy. Front Immunol 2023;14:1298571. 38162657 10.3389/fimmu.2023.1298571 PMC10757365

[114] Lee KL , Schlom J, Hamilton DH. Combination therapies utilizing neoepitope-targeted vaccines. Cancer Immunol Immunother 2021;70:875–85. 33033852 10.1007/s00262-020-02729-y PMC7979579

[115] Collins JM , Redman JM, Gulley JL. Combining vaccines and immune checkpoint inhibitors to prime, expand, and facilitate effective tumor immunotherapy. Expert Rev Vaccin 2018;17:697–705. 10.1080/14760584.2018.1506332 PMC8262093 30058393

[116] Yi M , Li T, Niu M, Zhang H, Wu Y, Wu K, . Targeting cytokine and chemokine signaling pathways for cancer therapy. Signal Transduct Target Ther 2024;9:176. 39034318 10.1038/s41392-024-01868-3 PMC11275440

[117] Waldmann TA . Cytokines in cancer immunotherapy. Cold Spring Harb Perspect Biol 2018;10:a028472. 29101107 10.1101/cshperspect.a028472 PMC6280701

[118] Lui G , Minnar CM, Soon-Shiong P, Schlom J, Gameiro SR. Exploiting an interleukin-15 heterodimeric agonist (N803) for effective immunotherapy of solid malignancies. Cells 2023;12:1611. 37371081 10.3390/cells12121611 PMC10297013

[119] Minnar CM , Lui G, Gulley JL, Schlom J, Gameiro SR. Preclinical and clinical studies of a tumor targeting IL-12 immunocytokine. Front Oncol 2023;13:1321318. 38260854 10.3389/fonc.2023.1321318 PMC10802843

[120] Lee KL , Benz SC, Hicks KC, Nguyen A, Gameiro SR, Palena C, . Efficient tumor clearance and diversified immunity through neoepitope vaccines and combinatorial immunotherapy. Cancer Immunol Res 2019;7:1359–70. 31292145 10.1158/2326-6066.CIR-18-0620 PMC8202970

[121] Fallon J , Tighe R, Kradjian G, Guzman W, Bernhardt A, Neuteboom B, . The immunocytokine NHS-IL12 as a potential cancer therapeutic. Oncotarget 2014;5:1869–84. 24681847 10.18632/oncotarget.1853 PMC4039112

[122] Smalley Rumfield C , Pellom ST, Morillon Ii YM, Schlom J, Jochems C. Immunomodulation to enhance the efficacy of an HPV therapeutic vaccine. J Immunother Cancer 2020;8:e000612. 32554612 10.1136/jitc-2020-000612 PMC7304848

[123] Fousek K , Horn LA, Qin H, Dahut M, Iida M, Yacubovich D, . An interleukin-15 superagonist enables antitumor efficacy of natural killer cells against all molecular variants of SCLC. J Thorac Oncol 2023;18:350–68. 36410696 10.1016/j.jtho.2022.11.008 PMC9974560

[124] Fabian KP , Padget MR, Donahue RN, Solocinski K, Robbins Y, Allen CT, . PD-L1 targeting high-affinity NK (t-haNK) cells induce direct antitumor effects and target suppressive MDSC populations. J Immunother Cancer 2020;8:e000450. 32439799 10.1136/jitc-2019-000450 PMC7247398

[125] Robinson TO , Schluns KS. The potential and promise of IL-15 in immuno-oncogenic therapies. Immunol Lett 2017;190:159–68. 28823521 10.1016/j.imlet.2017.08.010 PMC5774016

[126] Chiappinelli KB , Zahnow CA, Ahuja N, Baylin SB. Combining epigenetic and immunotherapy to combat cancer. Cancer Res 2016;76:1683–9. 26988985 10.1158/0008-5472.CAN-15-2125 PMC4873370

[127] Dan H , Zhang S, Zhou Y, Guan Q. DNA methyltransferase inhibitors: catalysts for antitumour immune responses. Onco Targets Ther 2019;12:10903–16. 31849494 10.2147/OTT.S217767 PMC6913319

[128] Jansz N , Faulkner GJ. Endogenous retroviruses in the origins and treatment of cancer. Genome Biol 2021;22:147. 33971937 10.1186/s13059-021-02357-4 PMC8108463

[129] Gruchot J , Herrero F, Weber-Stadlbauer U, Meyer U, Küry P. Interplay between activation of endogenous retroviruses and inflammation as common pathogenic mechanism in neurological and psychiatric disorders. Brain Behav Immun 2023;107:242–52. 36270439 10.1016/j.bbi.2022.10.007

[130] Goyal A , Bauer J, Hey J, Papageorgiou DN, Stepanova E, Daskalakis M, . DNMT and HDAC inhibition induces immunogenic neoantigens from human endogenous retroviral element-derived transcripts. Nat Commun 2023;14:6731. 37872136 10.1038/s41467-023-42417-w PMC10593957

[131] Hutchison S , Pritchard AL. Identifying neoantigens for use in immunotherapy. Mamm Genome 2018;29:714–30. 30167844 10.1007/s00335-018-9771-6 PMC6267674

[132] Noronha N , Durette C, Cahuzac M, E Silva B, Courtois J, Humeau J, . Autophagy degrades immunogenic endogenous retroelements induced by 5-azacytidine in acute myeloid leukemia. Leukemia 2024;38:1019–31. 38627586 10.1038/s41375-024-02250-6 PMC11073987

